# REM Sleep Behavior Disorder Is Not Associated with a More Rapid Cognitive Decline in Mild Dementia

**DOI:** 10.3389/fneur.2017.00375

**Published:** 2017-08-07

**Authors:** Luiza Chwiszczuk, Monica Haraldseid Breitve, Kolbjørn Brønnick, Michaela D. Gjerstad, Minna Hynninen, Dag Aarsland, Arvid Rongve

**Affiliations:** ^1^Department of Old Age Psychiatry, Helse Fonna HF, Haugesund Hospital, Haugesund, Norway; ^2^Department of Clinical Medicine, University of Bergen, Bergen, Norway; ^3^Department of Research and Innovation, Helse Fonna HF, Haugesund Hospital, Haugesund, Norway; ^4^Department of Psychiatry, Stavanger University Hospital, Stavanger, Norway; ^5^Norwegian Competence Center for Sleep Disorders, Haukeland University Hospital, Bergen, Norway; ^6^The Norwegian Centre for Movement Disorders, Stavanger University Hospital, Stavanger, Norway; ^7^Department of Neurology, Stavanger University Hospital, Stavanger, Norway; ^8^Department of Clinical Psychology, University of Bergen, Bergen, Norway; ^9^NKS Olaviken, Gerontopsychiatric Hospital, Bergen, Norway; ^10^Department of Old Age Psychiatry, Institute of Psychiatry, Psychology and Neuroscience, King’s College, London, United Kingdom; ^11^Centre for Age-Related Diseases (SESAM), Stavanger University Hospital, Stavanger, Norway

**Keywords:** REM sleep behavior disorder, sleep disorders, dementia, cognitive decline, longitudinal study, AD, dementia with Lewy bodies

## Abstract

**Objectives:**

REM sleep behavior disorder (RBD) is associated with cognitive dysfunctions and is a risk factor for development of mild cognitive impairment and dementia. However, it is unknown whether RBD is associated with faster cognitive decline in already established dementia. The main goal of this study was to determine if patients with mild dementia with and without RBD differ in progression rate and in specific neuropsychological measures over 4-year follow-up.

**Methods:**

This longitudinal, prospective study based on data from the DemVest study compares neuropsychological measures in a mild dementia cohort. A diagnosis of probable RBD (pRBD) was made based on the Mayo Sleep Questionnaire. Neuropsychological domains were assessed by Mini Mental State Examination, total score and figure copying, California Verbal Learning Test-II, Visual Object and Space Perception Cube and Silhouettes, Boston Naming Test, Stroop test, Verbal Category Fluency, Trail Making Test A and B.

**Results:**

Among the 246 subjects, 47 (19.1%) had pRBD at the baseline, and pRBD group was younger and with male predominance. During 4-year follow-up, we did not observe any significant differences in the rate of decline in neuropsychological measures. Patients with pRBD performed generally poorer in visuoconstructional, visuoperceptual, and executive/attention tests in comparison to RBD negative.

**Conclusion:**

We did not find any significant differences in progression rate of neurocognitive outcomes between dementia patients with and without RBD.

## Introduction

REM sleep behavior disorder (RBD) is a parasomnia, characterized by loss of normal muscle atonia in the REM sleep phase, accompanied by dream enactment. RBD was initially considered as an idiopathic disorder (iRBD); however, researches indicate that iRBD is a prodromal symptom of neurodegenerative disorders like Parkinson’s disease (PD), dementia in Parkinson’s disease (PDD), dementia with Lewy bodies (DLB), or multiple system atrophy (MSA), emerging after a period of 5–19 years. An observational study published by Schenck et al. showed that up to 81% of subjects with iRBD developed parkinsonian disorders or dementia after 16 years follow-up (1). Another study of Iranzo et al., from 2014, showed that estimated risk to develop neurodegenerative diseases was 90.9% after 14 years (2).

Several studies have shown that there are differences in cognitive performance in comparison with non-RBD controls. Most predominant are impairments in executive functions and attention (3–5), verbal episodic memory (4, 6), decision-making (7, 8), and visuospatial abilities (6, 9–11). Nevertheless, poorer visuospatial/visuoperceptual ability is debated, not all studies confirm this assumption (4, 6, 12, 13). Because there are clear similarities with cognitive profile in iRBD and those seen in alpha-synucleinopathies, it is proposed that this parasomnia can represent an early stadium of alpha-synucleinopathy-dependent neurodegeneration.

Idiopathic RBD is a known risk factor for developing mild cognitive impairment (MCI). In a population-based study, MCI developed in 32% of cognitively intact subjects with iRBD after a median of 3.8 year of follow-up with a 2.2-fold increased risk of developing MCI (14). In samples from sleep disorder centers, the risk is even higher, up to 50–65% compared to 8% in healthy subjects (13, 15, 16).

### RBD in Parkinson’s Disease

There have also been conducted some studies in Parkinson’s disease on patients with and without RBD. For RBD-positive PD patients, the frequency of MCI was estimated up to 73% (13). Concomitant RBD was found to be associated with poorer performance on tests measuring verbal memory, executive, and visuospatial functions, compared to patients without RBD (17). Data about frequency of developing PDD in RBD positive and negative patients are inconsistent. Postuma et al. demonstrated, in a 4-year follow-up observation, a clear higher risk of developing dementia in PD patients with concomitant RBD (18). There have also been published results reporting higher occurrence of dementia in RBD positive than RBD negative (42 vs. 7%) (19). Others, however, have suggested no differences among them (20, 21), and two longitudinal studies with 2- and 8-year follow-up period have not shown any worsening of general cognition based on Mini Mental State Examination (MMSE) (22, 23).

To the best of our knowledge, no study to date has systematically followed a cohort of RBD patients with mild dementia to determine cognitive deterioration over time, thus little is known about longitudinal changes in neuropsychological profiles in MCI and dementia with concomitant RBD.

There are only few studies assessing differences in progression of cognitive decline globally and in different cognitive domains over time but they are only focused on healthy RBD subjects or RBD subjects suffering from PD (24). In all previous studies, it was underlined the need to collect more data, larger samples sizes, and the need for longitudinal observations. With our study, we wish to fill this space and characterize dementia patients with coexistent RBD.

The purpose of this study was to assess longitudinally cognitive functions in a group of patients with mild dementia with and without probable RBD (pRBD). We wanted to answer the questions: do MCI and mild dementia patients with and without pRBD progress differently in cognition, measured by MMSE, and in specific cognitive domains over time? Our hypothesis is that presence of RBD can accelerate global dementia progression and more rapid deterioration in visuospatial, executive, and attention domains.

## Materials and Methods

### Sample and Procedures

From a total 261 participants registered in the DemVest study database (included between 2005 and 2013), 246 were included in our analysis. We excluded five with uncertain diagnoses, and those with alcoholic MCI/dementia. Participants were diagnosed with dementia according to DSM-IV. Patients were classified as having AD when fulfilling the criteria of National Institute and Neurological and Communicative Disorders and Stroke-Alzheimer’s disease and related Disorders (NINDS-ADRDA) (25). Diagnosis of DLB was made in accordance with the Third Report of the DLB Consortium: “Diagnosis and management of dementia with Lewy bodies” (26). PD was diagnosed according to the criteria of the United Kingdom Parkinson’s disease Society Brain Bank (27) and for PDD Emre’s criteria (28). Patients were followed annually, and after 2 and 5 years, a consensus panel of dementia experts re-evaluated the clinical diagnoses, based on all available information including neuropathologically verification for subset of 56 individuals (data not published yet).

### Ethical Issues

This study was carried out and approved in accordance with the recommendations of Norwegian Regional Ethics Committee and Norwegian authorities for collection of medical data. All subjects gave written informed consent in accordance with the Declaration of Helsinki.

### Sleep Assessment

Probable RBD diagnosis was assessed with the Mayo Sleep Questionnaire (MSQ) (29). The MSQ is a 16-item questionnaire specifically designed to detect sleep disturbances, using bed-partners as informants. Patients received a pRBD diagnosis when the core question: “Have you ever seen the patients appear to act out his/her dreams while sleeping (punched or flailed arms in the air, shouted, screamed)?” (Q1) was answered affirmatively. RBD might not be reported continuously even though persisting (22). “We, therefore, allocated all patients that reported RBD based on the core RBD questions of the MSQ at baseline or follow-up in the pRBD group. In longitudinal analysis where patients were RBD positive at just one or two follow-ups, they were allocated in pRBD at that exact point.”

### Neuropsychological Tests

Patients were assessed with a comprehensive neuropsychological test battery: MMSE, California Verbal Learning Test II, Boston Naming Test, Verbal Category Fluency, Stroop tests, Visual Object and Space Perception (VOSP) Silhouettes and Cube, Trail making Test A and B (TMTB). Details of selection and testing are described previously (21).

### Other

All patients were assessed with a structured interview, a general medical examination, blood samples, and in some cases cerebrospinal fluid samples. Parkinsonian symptoms were rated by the motor part of Unified Parkinson’s Disease Rating Scale (UPDRS) (30). MRI or CT was performed in order to exclude other conditions that could influence cognition. ^123^I-FP-CIT-SPECT (DATScan) was performed in some patients with suspected DLB. Medication analysis was performed to assess the possibility of drug-induced RBD. Diagnosis was neuropathological verified in 46 cases.

### Statistical Analysis

Statistical analyses were performed using SPSS IBM Software, v. 22.0. Clinical and demographic data at baseline were inspected with regard to distribution. If data conformed to the normal distribution, *t*-tests were used to assess between-group differences. For non-normally distributed data the non-parametric Mann–Whitney *U*-test was performed, and Chi-square test for categorical data.

Longitudinal analyses were performed using generalized linear mixed models (GLMM). In order to examine if pRBD was a predictor for more rapid dementia progression or a predictor for differential progression in neuropsychological domains, we set up a model with time (fu; entered as a categorical variable), pRBD (rbd) and the interaction term of fu*rbd as fixed effects. As confounders in the analyses, we entered age, education, and sex as covariates (fixed effects). Only subjects were set as random effect in order to correct for dependency of these clustered measures. The analyses were performed using the gamma distribution with a log link function for continuous variables and logit link function for binomial (dichotomous) variables. Variables from Stroop W, Stroop C, Stroop CW, and TMT A and TMT B were normally distributed and analyses for normal distribution were performed. We used Akaike Information Criterion to assess model optimization. All cognitive data were entered as raw scores.

## Results

Of the total of 246 subjects at the baseline, 122 (49.6%) participants were diagnosed with probable or possible AD, 10 (4.1%) with mixed AD and VaD, 83 (33.7%) with probable or possible DLB, 18 (7.3%) with PDD, 5 (2%) with FTD, and 8 (3.3%) with MCI.

The pRBD group had male predominance (30 men vs. 17 women, *p* = 0.001), was younger (*p* = 0.025, 95% CI 0.023, 0.029), had higher score on UPDRS motor scale (*p* = 0.004, 95% CI 0.004, 0.006), was more likely to use anti-parkinsonian medication (28.3% vs. 4.7%, *p* = 0.001), but no other types of medication that could influence on CNS or sleep (i.e., benzodiazepines, antidepressants, neuroleptics, melatonin). Detailed demographical and clinical data for RBD and non-RBD patients at baseline are presented in Table 1.

**Table 1:** Demographics and clinical characteristics of REM sleep behavior disorder (RBD) positive and negative groups at baseline.

	RBD (*n* = 47)	Non-RBD (*n* = 174)	*p*-Value (95% CI) or OR*
Age (years), SD	73.4 (7.5)	76.1 (7.6)	0.025 (0.023, 0.029)
Gender (M), %	30 (63.8)	66 (37.9)	0.001 (0.177, 0.676)*
Education (years)	9.8 (2.7)	9.7 (3.0)	0.443
MMSE, SD	23.8 (3.1)	23.6 (2.7)	0.515
Positive DATScan (*n* = 53), *n* (%)	17/23 (73.1)	22/30 (73.9)	0.607*
Dementia with Lewy bodies and dementia in Parkinson’s disease diagnosis, *n* (%)	37 (78.7)	52 (29.9)	<0.001 (3.443, 15.361)*
UPDRS III (SD)	12.7 (13.6)	7.0 (11.3)	0.004 (0.004, 0.006)
Parkinson’s medication, %	28.3	4.7	<0.001 (3.045, 20.645)*
Dementia medication, %	45.7	43.3	0.773*
Mean duration of RBD at baseline (years, SD)	6.91 (8.3)	–	–
CIRS total (SD)	6.52 (2.7)	5.63 (2.4)	0.051

*Chi-square.

*MMSE, Mini Mental State Examination; UPDRS, Unified Parkinson’s Disease Rating Scale; CIRS, Chronic Illness Rate Scale*.

**Figure 1:**
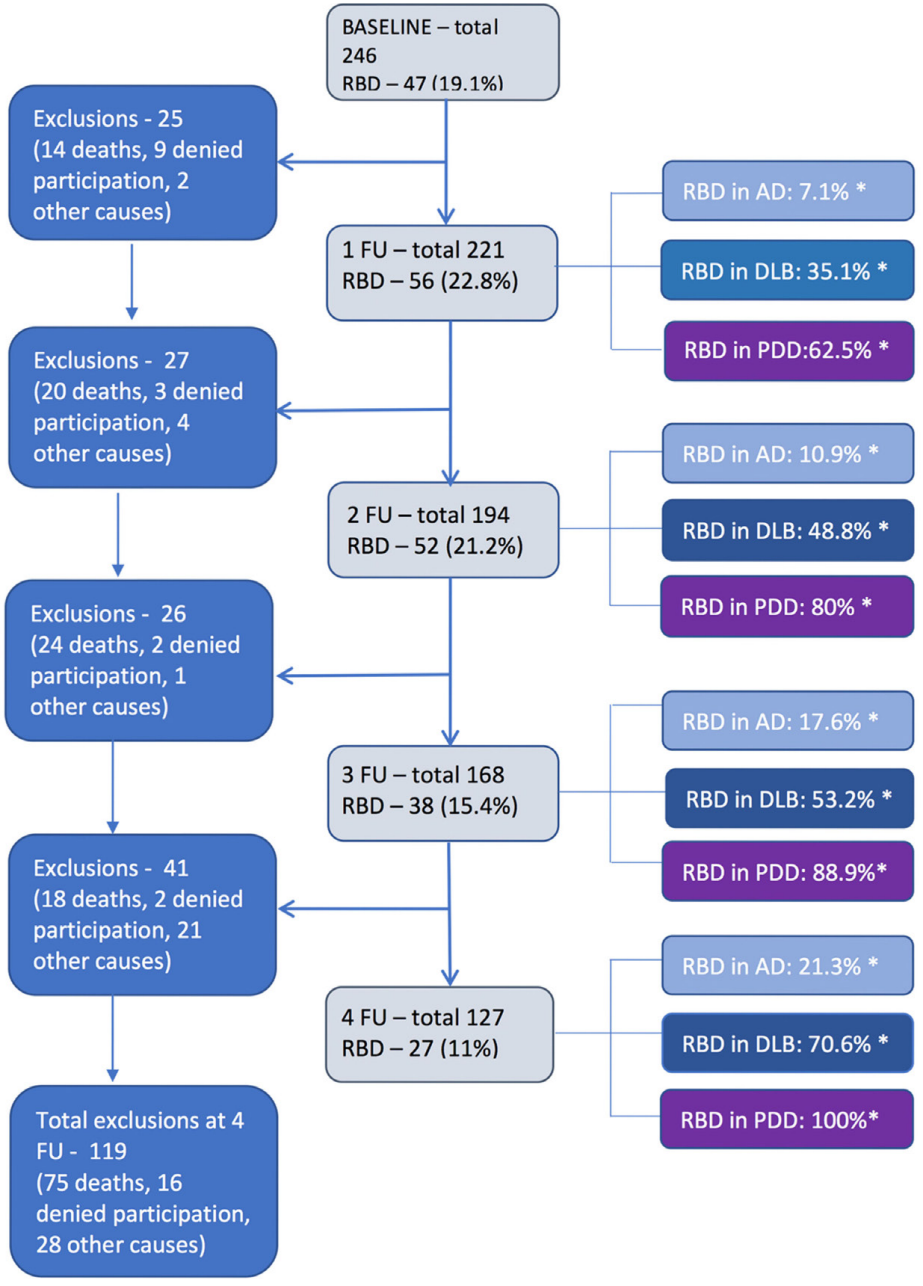
Number of subjects total and REM sleep behavior disorder (RBD) positive in each follow-up (*percentage of RBD positive patients within each diagnostic group; sum is not 100%).

Total number of subject and number of subjects with pRBD and percentage among diagnosis analyzed in each follow-up is presented in Figure 1.

Longitudinally, there was no statistically significant interaction effect between time and RBD vs. non-RBD regarding progression rates measured with MMSE or other neuropsychological tests corrected for age, sex, and education (see Table 2).

**Table 2:** Effects of probable RBD (pRBD) and interaction between pRBD and time in dementia cohort (shown only statistically significant findings).

	pRBD				Interaction pRBD*fu
	Main effect (*F*, *p*)	pRBD (+), fixed coefficient Est (SD), *t*	*p*	95% CI	Main effect (*F*, *p*)
MMSE pentagon	8.002, 0.005	−1.83 (0.7), −2.613	0.009	−3.203, −0.454	1.231, 0.297
VOSP Cube	16.94, <0.001	−0.31 (0.13), −2.441	0.015	0.006, 0.556	1.335, 0.256
TMT A	18.44, 0.001	82.2 (29.98), 2.742	0.006	−141.01, −23.32	1.327, 0.259
	9.11, 0.003^a^	57, 38, 31.22, −1.838^a^	0.067^a^	−118, 74, 3.99^a^	1.553, 0.187^a^
Stroop W	16.11, <0.001	−12.42 (5.71), −2.172	0.030	1.186, 23.651	0.26, 0.904

*^a^Corrected for Unified Parkinson’s Disease Rating Scale*.

*MMSE, Mini Mental State Examination; VOSP, Visual Object and Space Perception; TMTA, Trail Making Test A; Stroop W, Stroop Words*.

**Figure 2:**
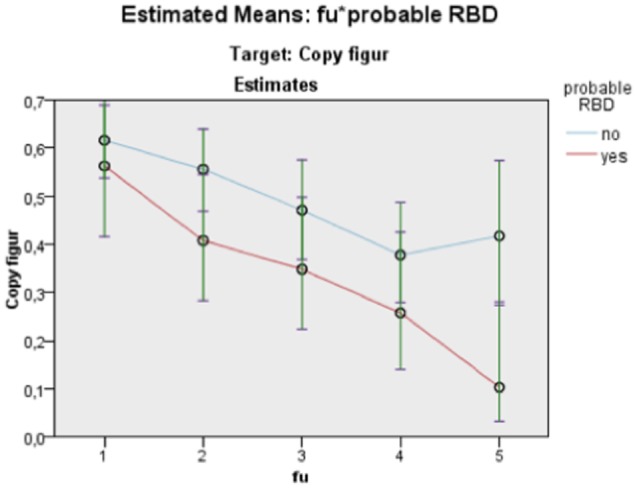
Estimated means: fu*probable REM sleep behavior disorder (RBD). Target: Mini Mental State Examination (MMSE) copy figure. Continuous predictors are fixed at the following values: BL_EDUCATION = 9.7, age = 76.

**Figure 3:**
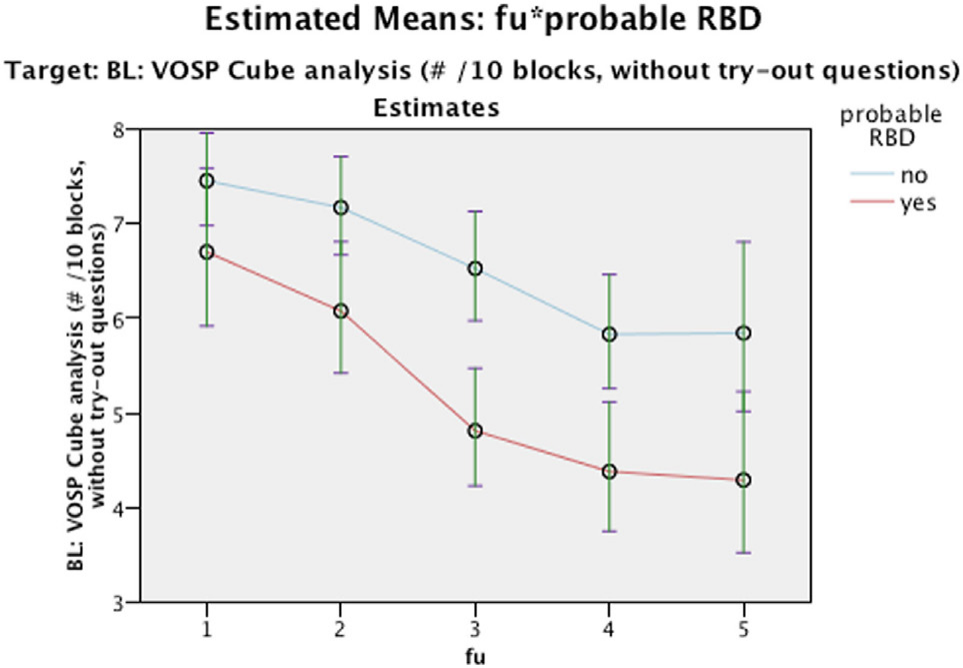
Estimated Means: fu*probable REM sleep behavior disorder (RBD). Target: BL: visual object and space perception (VOSP) cube analysis (#/10 blocks, without try-out questions). Continuous predictors are fixed at the following values: BL_EDUCATION = 9.7, age = 76.

**Figure 4:**
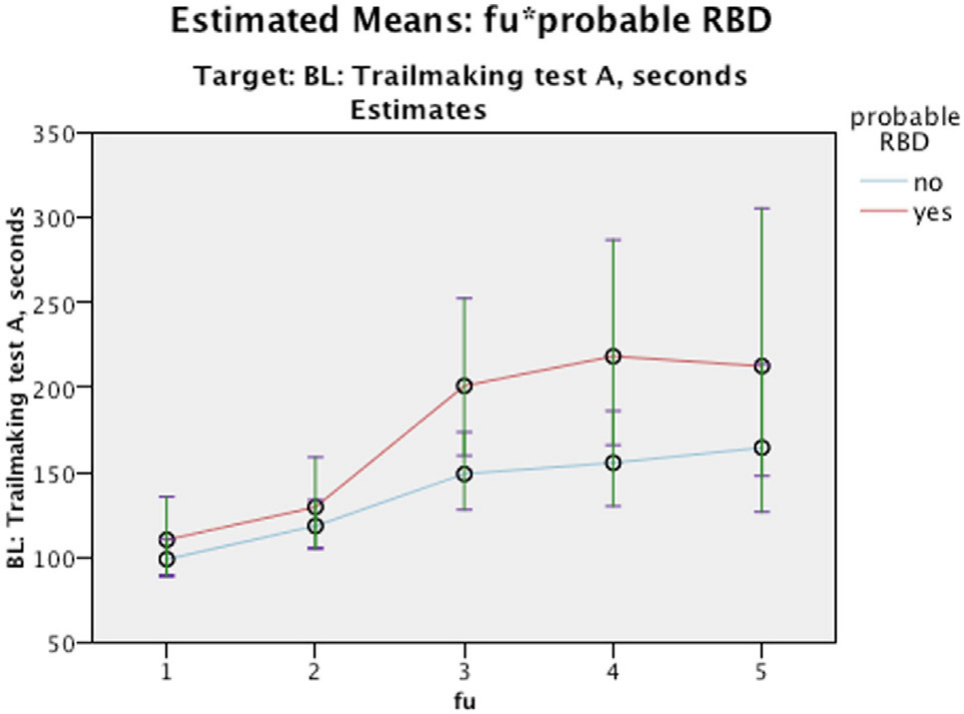
Estimated Means: fu*probable REM sleep behavior disorder (RBD). Target: BL: Trailmaking test A, seconds. Continuous predictors are fixed at the following values: BL_EDUCATION = 9.8, age = 76.

**Figure 5:**
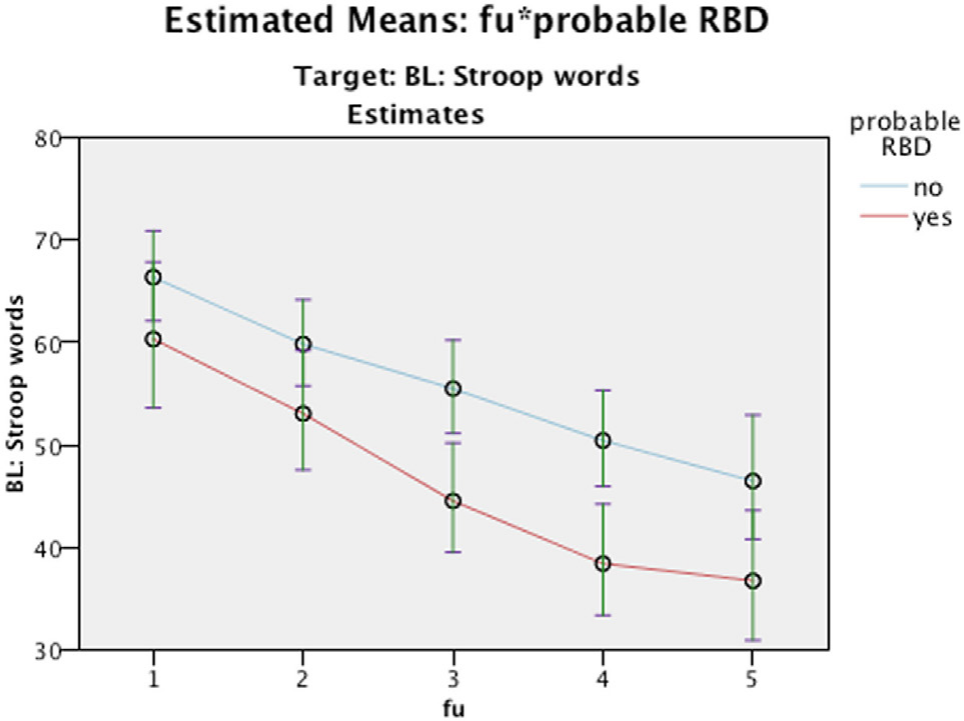
Estimated Means: fu*probable REM sleep behavior disorder (RBD). Target: BL: Stroop Words. Continuous predictors are fixed at the following values: BL_EDUCATION = 9.7, age = 76.

Nevertheless, it is worth to note that even though we did not find any interaction differences between time and cognition, the RBD positive group performed significantly poorer in MMSE figure copying, VOSP Cube, TMT A, and Stroop Words in comparison to RBD negative as seen in main effect (see Figures 2–5). Due to bradykinesia and bradyphrenia, one of the main symptoms in PDD and DLB, and possible confounders on tests Stroop tests, we controlled it for UPDRS scores. This did not change the results, except from TMT A, where performance time no longer were statistically different between pRBD-positive and -negative subjects.

Because our cohort was heterogeneous within diagnosis, we also analyzed the AD group separately to see if there were any differences. The DLB group was too small for such statistical analyses. Patients with AD and positive RBD performed poorly on MMSE and Stroop Word in comparison with RBD negative patients, and had statistically significant more rapid decline in VOSP Cube (see Table 3).

**Table 3:** Effects of probable RBD (pRBD) and interaction between pRBD and time in AD cohort (*n* = 122) (shown only statistically significant findings).

	pRBD				Interaction pRBD*fu			
	Main effect (*F*, *p*)	Fixed coefficient Est (SD), *t*	*p*	95% CI	Main effect (*F*, *p*)	Fixed coefficient Est (SD), *t*	*p*	95% CI
MMSE	4.214, 0.041	−0.30 (0.09), −3.27	0.001	0.121, 0.485	1.231, 0.297	–	–	
VOSP Cube	9.05, 0.003	−0.28 (0.19), −1.47	0.141	–	4.697, 0.001	−3.04 (1.23), −2.471	0.014	−4.087, −0.074
Stroop W	16.11, <0.001	−12.42 (5.71), −2.17	0.03	1.186, 23.651	0.26, 0.904	–	–	

*MMSE, Mini Mental State Examination; VOSP, visual object and space perception; TMTA, Trail Making Test A; Stroop W, Stroop Words*.

## Discussion

The main finding in this study was that there were no differences in rate of cognitive decline measured with MMSE in dementia patients with or without pRBD. Similarly, patients in both groups declined equally in all neuropsychological tests measuring short and delayed verbal memory, verbal fluency, executive functions, and visuospatial abilities during 4 years of observation.

Since many studies have shown that iRBD is a risk factor for developing cognitive problems, neurodegenerative disorders and can accelerate cognitive dysfunction in PD, one could expect a faster progression of cognitive deficits in the RBD-positive dementia group. In a very similar, longitudinal study on PD in early stages, it is postulated that RBD could reflect more severe neurodegeneration and contribute to greater rates of cognitive decline (24). However, our data do not support the hypothesis that the occurrence of RBD is associated to an accelerated dementia progression.

Lack of differences in progression speed can be explained by the hypothesis that subtle differences in more advanced stadiums of cognitive impairment disappear as a consequence of reduction in multiple cognitive domains and overlapping pathology, but we did not observe such trend in earlier follow-ups where dementia was not very advanced. This suggests that RBD may be a part of established, more advanced neurodegeneration, and probably not associated with different rate of progression at this stage of dementia generally. There are, so far, more evidences for that synucleinopathy probably proceeds RBD, than the opposite. Previously published studies in iRBD have shown reduction in dopamine transporters striatal uptake before appearance of other extrapyramidal symptoms (31, 32). Moreover, iRBD patients often present other markers of alpha-synucleinopathy such as hyperechogenicity of the substantia nigra or hyposmia (33). Furthermore, two autopsy case studies in iRBD patients revealed pathology consistent with alpha-synucleinopathy (34, 35). Therefore, RBD may be considered as a marker of alpha-synucleinopathy rather than primary risk factor, which influences progression rate itself (36). Similar hypothesis is mentioned in a study of Manni et al. (12).

Furthermore, RBD is also highly predominant in MSA, with a prevalence of 88% in a recent study by Palma (37). Thus, almost all MSA patients should develop dementia, but dementia is not as common in MSA (15%) as in other synucleinopathies (PDD or DLB) (38). This fact supports again the hypothesis that RBD is an additional feature or consequence of alpha-synucleinopathy, rather than a risk factor for cognitive impairment itself.

On the other hand, RBD could theoretically have negative effect on cognition as sleep affecting pathology. REM sleep is important for memory consolidation and stabilization (39). Reduction in REM sleep leads to impairment in all learning phases and memory formation (40). However, several studies show that RBD is not necessary related to lack of REM sleep (11, 20), so REM sleep deprivation does not seem to be the whole explanation for impaired cognition in RBD.

As also shown in previous studies, we found that RBD-positive patients had in general lower average scores in tests measuring visuospatial abilities and executive functions (MMSE pentagon, VOSP Cube, TMT A and Stroop Words), which corresponds with neuropsychological profile seen in alpha-synucleinopathies. Reduced processing speed in TMT A is probably related to presence of more advanced parkinsonian symptoms in the RBD group, since after controlling for the UPDRS score, there were no longer any significant differences. Poorer performances in pRBD in executive functions/attention and visuospatial abilities from the beginning indicate earlier and longer duration of pathology, because our patients with pRBD have had longer duration of dementia symptoms from debut to baseline.

The RBD-positive AD patients had a faster decline on VOSP Cube. It was not found in other tests tapping the same function, and it could be due to type I error. Therefore, this needs confirmation in future studies before taken as a fact.

### Limitations and Advantages

A major limitation of our study was lack of polysomnography validation of RBD. Diagnosis of pRBD was based on bed-partner report on MSQ and, therefore, only anamnestic, but sensitivity and specificity of MSQ in diagnosing pRBD are estimated to 98 and 74%, respectively (29). It should be noted that MSQ can both underestimate, if informant is not aware of sleep problem, or overestimate, if obstructive sleep apnea symptoms mimic RBD. In our study group, near 50% of the informants reported that their relatives with dementia also had OSA symptoms (snoring or breath stops). Boot et al. suggest that MSQ only has 0.66 positive predictive value, which means that even more patients could have RBD (14). In all dementia types, prevalence of RBD is estimated to 18.5% (41). Prevalence of pRBD in our dementia cohort was 19.1% (studies share partially same group of participants), but true prevalence of RBD in dementia is not yet known. We mainly found negative results in this study and have not adjusted the alpha level, even though we have performed multiple analyses. Positive findings could, thus, hypothetically be a result of coincidence.

To the best of our knowledge, this study is the first to shed light on the question of how the presence of RBD in dementia influences cognitive performance, dementia progression, and cognitive changes over time. In addition, it is one of the largest cohorts of RBD patients with longitudinal follow-up. Our patients were referred to a memory clinic, not a sleep center, thus a selection bias leading to overrepresentation of pRBD is unlikely. The current study cohort was assessed regarding RBD inducing medication and no such association was found (42). Structural imaging (CT or MRI) was also performed in all participants, which made it possible to exclude subjects with major structural lesions that could secondarily lead to RBD. Not all previous studies have used such exploration. The statistical method, GLMM, has a clear advantage in this kind of longitudinal study with multiple follow-up points.

### Conclusion and Perspectives

Despite several studies having shown cognitive differences between “RBD sufferers” and controls without RBD, our findings did not indicate that pRBD is a risk factor for faster cognitive decline in established dementia. Still, little is known about how presence of RBD influences cognition and in what way. Further longitudinal studies on larger groups are needed, both in healthy subjects and patients who have developed a neurodegenerative condition, and with standardized batteries of neuropsychological assessment.

## Ethics Statement

This study was carried out and approved in accordance with the recommendations of the Norwegian Regional Ethics Committee and Norwegian authorities for collection of medical data. All subjects gave written informed consent in accordance with the Declaration of Helsinki.

## Author Contributions

LC—conception, writing (drafting) of the manuscript, statistical analysis. MB—writing (drafting) of the manuscript. KB—statistical analysis. MG, AR, DA, MH—organization, direction, and editing of the manuscript. DA—chef of the DEMVEST project, database responsible.

## Conflict of Interest Statement

The authors declare that the research was conducted in the absence of any commercial or financial relationships that could be construed as a potential conflict of interest.
